# Profiling of porcine B-cell receptor heavy-chain repertoires indicates the development of a wide public pseudorabies virus-specific immune response after vaccination and challenge

**DOI:** 10.1093/discim/kyag009

**Published:** 2026-05-05

**Authors:** Valentin Herbet, Edouard Hirchaud, Yannick Blanchard, Véronique Beven, Léon-Charles Tranchevent, Michael Jarman, Bharti Mittal, Céline Deblanc, Frédéric Paboeuf, John A Hammond, Nicolas Eterradossi, Daniel Dory, Marie Bonnet-Di Placido

**Affiliations:** ANSES, Ploufragan-Plouzané-Niort Laboratory, VIRPIG Unit (Swine Virology, Innovation & Genomics Unit), 41 rue de Beaucemaine, Ploufragan 22 440, France; UFR of Life Sciences Environment, University of Rennes, Rennes 35700, France; ANSES, Ploufragan-Plouzané-Niort Laboratory, VIRPIG Unit (Swine Virology, Innovation & Genomics Unit), 41 rue de Beaucemaine, Ploufragan 22 440, France; ANSES, Ploufragan-Plouzané-Niort Laboratory, VIRPIG Unit (Swine Virology, Innovation & Genomics Unit), 41 rue de Beaucemaine, Ploufragan 22 440, France; ANSES, Ploufragan-Plouzané-Niort Laboratory, VIRPIG Unit (Swine Virology, Innovation & Genomics Unit), 41 rue de Beaucemaine, Ploufragan 22 440, France; ANSES, Ploufragan-Plouzané-Niort Laboratory, VIRPIG Unit (Swine Virology, Innovation & Genomics Unit), 41 rue de Beaucemaine, Ploufragan 22 440, France; The Pirbright Institute, Pirbright, Woking GU24 0NF, UK; The Pirbright Institute, Pirbright, Woking GU24 0NF, UK; ANSES, Ploufragan-Plouzané-Niort Laboratory, VIRPIG Unit (Swine Virology, Innovation & Genomics Unit), 41 rue de Beaucemaine, Ploufragan 22 440, France; ANSES, Ploufragan-Plouzané-Niort Laboratory, SPPAE Service (SPF Pig Production and Experimentation service), 41 rue de Beaucemaine, Ploufragan 22 440, France; The Pirbright Institute, Pirbright, Woking GU24 0NF, UK; ANSES, Ploufragan-Plouzané-Niort Laboratory, 41 rue de Beaucemaine, Ploufragan 22 440, France; ANSES, Ploufragan-Plouzané-Niort Laboratory, VIPAC Unit (Virology, Immunology and Parasitology in Poultry and Rabbits Unit), 41 rue de Beaucemaine, Ploufragan 22 440, France; The Pirbright Institute, Pirbright, Woking GU24 0NF, UK

**Keywords:** porcine antibody repertoire, heavy-chain BCR, bulk sequencing, pig, public antibodies, convergence

## Abstract

**Introduction:**

The antibody repertoire reflects the immune history of an individual. In the present study, we characterized the porcine antibody heavy-chain repertoire following vaccination and challenge with pseudorabies virus (PRV). Due to the limited number and diversity of porcine germline V-genes, we sought to also identify and quantify the public antibody response to different strains that could indicate conserved epitopes.

**Methods:**

Three groups, each with four specific pathogen-free pigs were vaccinated twice with the Bartha K61 strain and subsequently challenged twice, each group with a different PRV strain. Longitudinal blood sampling was performed before immunization and at multiple time points chosen to coincide with expected peaks of antigen-specific B cell clonal expansion. Bulk heavy-chain repertoire sequencing was carried out and analysed using the Immunoglobulin Multi-species Annotation Tool, and heavy-chain amino acid sequences were clustered. This approach allowed the identification of B-cell receptor (BCR) populations sharing more than 96% identity in their complementary determining regions, suggesting similar pathogen recognition capabilities.

**Results:**

By selecting clusters with frequency kinetics matching immunization or infection events, we identified numerous public clusters shared between pigs within and across groups. Notably, several clusters were detected in more than eight animals, and two clusters were shared by all twelve pigs.

**Conclusion:**

Although the selected clusters represent only a subset of the putative PRV-induced repertoire, these findings reveal a striking degree of convergence in the antibody response to PRV in pigs. This work demonstrates that cluster kinetics analysis of bulk BCR sequencing data can identify candidate antigen-specific antibody heavy-chain lineages and provides a framework for future discovery of PRV-specific antibodies.

## Graphical Abstract

**Graphical Abstract:**
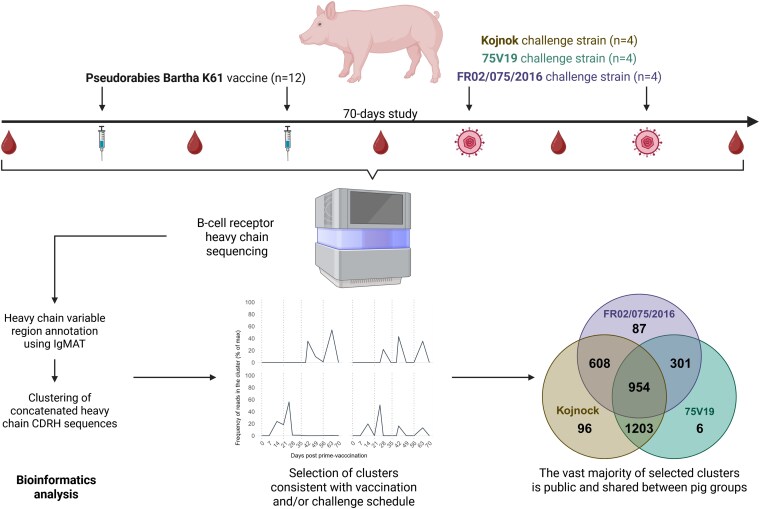
Created in BioRender. Biorender, V. (2026) https://BioRender.com/07sbe1y.

## Introduction

Pigs are an essential source of food globally, but their production is challenged by numerous pathogens that impair zootechnical performance and pose sanitary risks. Diseases such as Aujeszky's disease, influenza, porcine reproductive and respiratory syndrome, African swine fever, and porcine epidemic diarrhoea remain insufficiently controlled worldwide [1]. Antibodies are major effectors of the adaptive immune response, and their study frequently guides the development of vaccines and serological diagnostic tools [2–4] that are essential for disease management. Membrane-bound antibodies expressed at the surface of B cells are referred to as B-cell receptors (BCRs). After maturation, B cells differentiate into plasma cells capable of secreting large amounts of pathogen-specific antibodies [5].

Pig antibodies are composed of two identical heavy and light chains, respectively, each comprising a constant and a variable region. Constant regions define antibody isotypes, which directly influence their effector functions [6]. Pigs express five immunoglobulin heavy-chain isotypes (IgG, IgA, IgM, IgE, and IgD), corresponding to the constant regions Cγ, Cα, Cµ, Cε, and Cδ, respectively, as well as two light-chain isotypes, λ and κ, encoded by Cλ and Cκ [7]. Moreover, pigs express six IgG sub-types (IgG1 to IgG6), of which IgG5 and IgG6 comprise two allotypes (namely IgG5-1, IgG5-2 and IgG6-1, IgG6-2). Variable regions are encoded by the V, D, and J gene segments for the heavy chain, and by the V and J gene segments for the light chain, forming the framework regions (FR1-FR4) and the complementarity-determining regions (CDR1-CDR3). The CDR regions are hypervariable and form the primary contact with the epitope [8]. To attain high specificity against a wide range of epitopes, B cells rely on several diversification mechanisms. Immature B cells contain multiple V, D, and J gene segments within their immunoglobulin loci. During V(D)J recombination, one segment of each type (V, D, and J) is randomly selected and joined, generating antibody diversity through combinatorial diversity and junctional diversification. This process occurs in both heavy and light chains, and random pairing between heavy and light chains further expands repertoire diversity. In addition, somatic hypermutation (SHM), mediated by activation-induced cytidine deaminase (AID), introduces point mutations within the variable region and further diversifies antigen specificity [9]. Pigs exhibit novel features in this diversification process when compared with human and model species such as mouse. Indeed pigs possess fewer V, D, and J gene segments (23 V, 4 J and 5 D) [7], which limits the number of possible V(D)J combinations by around 45 and 75 times [10] compared to the human and murine genetic loci, respectively, that are compensated by around 100 times higher SHM rates (pigs SHM rates resolves around 30 mutations/kb) that maintains a comparable level of circulating antibody diversity [11, 12].

The BCR repertoire is defined as the population of antibodies expressed on B-cells by an individual at a given time point in a given tissue. When assessed longitudinally, repertoire dynamics allow for the identification of pathogen-specific BCR populations by comparing pre- and post-exposure states [13–15]. At first, BCR repertoires were characterized through H-CDR3 length distributions [16–18], a method that provided an overview of diversity but did not allow access to the millions of unique BCR sequences present in an individual. In humans, the total circulating B-lymphocyte repertoire is estimated at 9–17 million distinct clones [19], whereas similar estimations are not yet available for pigs. The development of next-generation sequencing (NGS) now enables access to large numbers of BCR sequences and facilitates the identification of V(D)J combinations that emerge following antigen exposure [20–22]. To increase the likelihood of capturing antigen-induced BCRs, sampling must occur around the peak of antigen-specific clonal expansion, typically between 3 and 9 days post-boost immunization, or within two weeks post-infection [23, 24]. In humans, convergent or public BCR sequences have been repeatedly shown to correspond to antigen-specific antibody responses following vaccination or infection, supporting their use as markers of pathogen-driven humoral immunity [25–27]. In pigs, the identification of antigen-specific BCR sequences has been challenging because of the above-mentioned specificities in generating BCR diversity. Only minor variations could be identified in the V, D, and J genes expression following vaccination or infection [22].

Pseudorabies virus (PRV) is a member of the family *Herpesviridae*, subfamily *Alphaherpesvirinae*, genus *Varicellovirus, species Suid alphaherpesvirus 1*. PRV is a neurotropic enveloped virus that is the causal agent of Aujeszky's disease and is lethal to piglets and induces abortion in pregnant sows. In older pigs, it causes neurological disorders, respiratory distress, hyperthermia, and significant weight loss with disastrous economic consequences. Eleven glycoproteins are present on the outer surface of the viral envelope (glycoproteins B, C, D, E, G, H, I, K, L, M, and N) [28]. Several of these glycoproteins play important roles during viral infection. For example, gB, gD, gH, and gL are involved in virus entry into the host cell. Glycoprotein B is also engaged into cell to cell and neuronal cell spreads. PRV induces a strong immune response following vaccination or challenge [29, 30], and its major immunogenic proteins, particularly glycoproteins B and E, are well documented. Their genomic sequences are conserved across strains and display minor differences (as described in Supplementary Figure S1). The Bartha K61 strain is a live attenuated ΔgE vaccine enabling the serological differentiation between infected and vaccinated animals (a DIVA strategy), based on the principle that only pigs infected with wild-type virus will produce anti-gE antibodies, whereas vaccinated pigs do not [29]. PRV is also known to induce strong immune responses, which leads to the development of PRV-specific antibodies. We exploited these features to explore pig BCR repertoires in a PRV vaccine-challenge animal experiment. Pigs received two consecutive doses of Bartha K61 vaccine, followed by two sequential challenges with one of three distinct pathogenic PRV strains. This repeated antigenic exposure boosted the immune response and enabled longitudinal analysis of BCR heavy-chain kinetics in relation to vaccination and challenge. This strategy enables the exploration of vaccination-related BCR populations and the identification of heavy chain sequences specific to antigens introduced by the challenge strains. Repeated vaccinations and challenges further allowed the emergence and development of both immunodominant BCR and non-immunodominant vaccine- or challenge-induced BCR sequences. Weekly blood sampling over 70 days allowed kinetic profiling of porcine BCR heavy-chain repertoires. We assessed whether BCR heavy-chain populations consistent with the vaccine and challenge schedule could be identified using a clustering strategy applied to variable region amino acid sequences annotated using the Immunoglobulin Multi-species Annotation Tool (IgMAT) suite [31]. BCR populations were grouped through the clustering of their heavy chain variable regions by amino acid sequence similarity. This clustering strategy lies on the hypothesis that BCR with similar sequences may recognize the same epitopes. We hypothesized that BCR heavy chain clusters whose temporal dynamics matched vaccination and challenge events would include PRV-induced BCR populations. Particular focus was given to public heavy chains, defined as sequences shared across animals, as we investigated whether their convergence might be influenced by repeated vaccination and challenge regimen with different PRV strains with some conserved antigenicity.

## Materials and methods

### Experimental design

All animal experiments were performed in agreement with French regulations on animal welfare and were approved by the National Committee for Ethics in Animal Experimentation ANSES/ENVA/UPEC (approval no.23-035#41781) and the French Ministry of Higher Education and Research (authorization number: APAFIS #41781-2023031710164483 v5).

**Figure 1:**
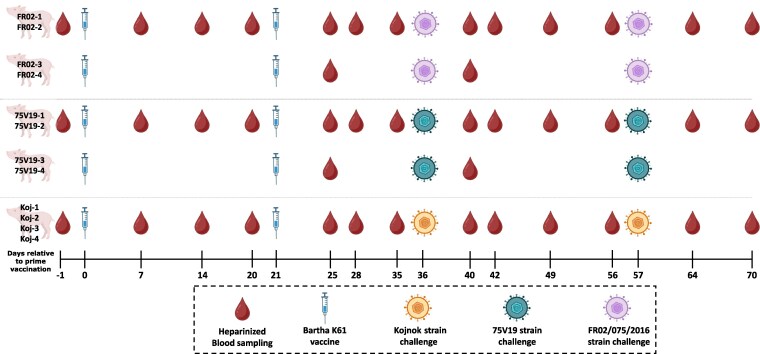
Experimental design of the PRV vaccination and challenge study. Twelve specific pathogen-free pigs were vaccinated intramuscularly twice with the Bartha K61 vaccine (days 0 and 21), then challenged oronasally twice (days 36 and 57) with one of three pathogenic PRV strains: Kojnok (*n* = 4), 75V19 (*n* = 4), or FR02/075/2016 (*n* = 4). Animals were housed in three independent containment rooms according to challenge strain. Longitudinal blood sampling was performed at multiple time points throughout the study to monitor serology and B-cell receptor repertoires. Additional tissue sampling was performed at necropsy as indicated. *Created in BioRender*. Biorender, V. (2026) https://BioRender.com/yuah2lp.

Twelve 56-day-old specific pathogen-free (SPF) large white pigs (source Anses-Ploufragan, France) were divided into three groups (*n* = 4) and housed in three independent A3 containment rooms (Fig. 1). Based on previous evaluations of vaccines against PRV, 4 pigs per group are sufficient to discriminate the vaccinated group from the non-vaccinated one [32]. This design allows robust detection of reproducible immune features while adhering to the principles of the 3Rs (Replacement, Reduction, and Refinement). The 12 pigs were derived from two separate litters, each consisting of six animals. Intramuscular vaccination of all 12 animals was performed at days 0 and 21 with the inactivated vaccine, AUSKIPRA®BK from HIPRA [33] (2ml per dose), containing the Bartha K61 strain [29] (GenBank accession number: JF797217.1). Each group was subsequently challenged oronasally (a total of 1.5 ml was administered per nostril and 2 ml into the oral cavity using a 5 ml syringe) at days 36 and 57 with one of three PRV strains (10^5^ TCID50/pig): Kojnok [34], this virulent strain was used as an immunizing strain for production of monoclonal antibodies, 75V19 [35], this strain was isolated in Belgium from a severe outbreak of pseudorabies, with nervous signs in pigs, respiratory illness in fattening swine and abortion in sows, or FR02/075/2016 [36], isolated from a dog that had died of PR. This probably traced back to a PRV strain circulating in wild boars. The 21 days delay between both challenges allowed the immune system to resolve the possible infection before being presented with pathogenic strains a second time, allowing for the boost of the immune system in healthy conditions. As the first 75V19 challenge did not elicit any reaction in pigs, the second challenge was done with a dose of 10^7^ TCID50/pig. All PRV strains were tested for mycoplasma and only the Kojnok strain elicited a Ct value of 35.4 evaluated by real-time PCR (Venor®GeM, qEP kit, Minerva Biolabs). Hereafter, animals are referred to as Koj-1 to Koj-4, 75V19-1 to 75V19-4, and FR02-1 to FR02-4. Siblings were evenly distributed across the challenge groups. As previously described [37], the SPF status of the pigs was checked at birth for a wide range of common pig pathogens.

Rectal temperatures and weights were monitored daily and weekly, respectively. Heparinized and clotted blood samples were collected at the following time points: D0, D7, D14, D20, D28, D35, D42, D49, D56, D64, and D70. Two additional heparinized blood samples were collected at D25 and D40. Final clotted samples were collected at D76, except for the 75V19-challenged pigs. Tonsils and trigeminal ganglia were collected from all pigs for later detection of PRV by qPCR.

### Detection of circulating antibodies against PRV gB and gE

To assess total circulating antibodies against PRV gB and gE, ID Screen® Aujeszky gB Competition and ID Screen® Aujeszky gE Competition (Innovative Diagnostics—IDVET, France) ELISAs were performed, respectively, according to the manufacturer's instructions. Briefly, serum samples were added to either gB- or gE-coated plates, incubated, and washed before addition of the corresponding anti-gB or anti-gE conjugate. Detection was performed using a TMB substrate, and optical density was read at 450 nm (OD450). Results were expressed as signal-to-noise ratios (S/N%), with positivity thresholds of <40% for gB and <60% for gE, as defined by the manufacturer's instructions.

### Real-time PCR detection of PRV from tonsil and trigeminal ganglion samples

As previously described [38], DNA was extracted from tonsil and trigeminal ganglion samples from all pigs using the ADIAMAG 200R RNA and DNA extraction kit with magnetic beads (BioX- Diagnostics, France), following manufacturer's instructions. The presence of PRV was then assessed using ADIAVET® PRV REALTIME kit (BioX- Diagnostics, France) based on the detection of the gene encoding for the PRV-gD, following the manufacturer's recommendations.

### Peripheral blood mononuclear cell (PBMC) isolation

PBMCs were isolated using Ficoll-Paque Plus (Sigma-Aldrich, Merck, Germany) immediately after heparinized blood collection, as previously described [30]. Following isolation, cells were stained with acridine orange and counted using the Luna-FL Dual Fluorescence Cell Counter (Logos Biosystems, Korea). A total of 2.10^6^ cells were resuspended in 250 µl of DPBS and 750 µl of TRIzol LS (ThermoFisher Scientific, USA), and stored at −80°C until further use.

### Amplification and library preparation of immunoglobulin heavy-chain variable regions

After thawing, RNA was extracted from frozen PBMCs in TRIzol LS according to the manufacturer's instructions and eluted in 25 µl of ultrapure water. cDNA was synthesized from extracted RNA using SuperScript™ IV (SSIV) Reverse Transcriptase (ThermoFisher Scientific, Invitrogen, Lithuania). Briefly, 2 µl of RNA was mixed with 1 µl of 4 µM random primers (Takara, Japan), 1 µl of 10 mM dNTP mix (Promega, USA) and 8 µl of nuclease-free water, and incubated for 5 minutes at 65°C. After cooling on ice for 2 minutes, 2 µl of 25 mM MgCl_2_ (Promega, USA), 4 µl of SSIV 5× buffer, 1 µl of DTT (0.1 M) and 1 µl of SSIV enzyme were added. The mix was then incubated for 15 minutes at 25°C, 60 minutes at 50°C, and 15 minutes at 70°C.

Variable regions of heavy chains (of the IgM, IgG, and IgA isotypes) were amplified separately for each animal and time point. Sequencing protocols previously developed for cattle [39] and pigs [40] were adapted for the pig whole BCR repertoire in this study. Briefly, variable regions were amplified through three consecutive rounds of PCR. PCR 1 amplified the immunoglobulin variable regions, PCR 2 added Illumina adapter overhangs, and PCR 3 added unique Illumina index pairs to identify each sample, using adaptor sequences as anchors. Reaction mixes, amplification conditions, and primer sequences are described in Supplementary Table S1.

PCR products were purified after PCR 2 and PCR 3 using Ampure XP magnetic beads (Agencourt-Beckman, USA). PCR amplicons were mixed with magnetic beads at a 1.8 ratio (20 µl DNA added to 36 µl bead solution) and purified as recommended by the manufacturer.

Finally, DNA concentrations were quantified using Quant-iT^TM^ dsDNA Broad-Range Assay Kit (ThermoFisher, Invitrogen, USA). For each sample, 2 µl of DNA was mixed with 1 µl of Quant-it dsDNA BR reagent and 199 µl of Quant-it dsDNA BR buffer. DNA samples, reaction mixes, and dsDNA standard solutions were plated, and fluorescence was measured using TECAN NanoQuant Plate™ reader.

### Next-generation sequencing of immunoglobulin heavy-chain amplicons

Two sequencing runs on two separate PCR amplification products were performed to limit the introduction of bias due to PCR amplifications [41]. For each run, the three amplified isotype amplicons (IGHG, IGHM, IGHA) were pooled at 4 nM. Sequencing libraries were loaded at 12 pM into a MiSeq flow cell using the Reagent Kit v2 (Illumina) for paired-end sequencing (500 cycles, 2×250) on a MiSeq sequencer.

### Immunoglobulin heavy-chains repertoire analysis

Fastq files generated by Illumina sequencing were first assessed for quality metrics including GC content, sequence duplication levels and overall read quality using the FastQC v0.11.5 visualization tool [42]. Based on this initial assessment, high-quality reads were selected and processed with TrimGalore 0.6.10 [43] for adaptor removal trimming of low-quality 3’ bases using a Phred score cut-off of 30 (equivalent to 99.9% accuracy). Forward and reverse paired-end reads were then merged using FLASH (Fast Length Adjustment of Short reads) v1.2.11 [44] with default parameters (minimum overlap 10 bp). Constant regions and V(D)J gene segments were identified using, respectively, BLAST 2.12.0+ and IgBLAST 1.22.0 [45]. Due to the high similarity between pig antibody genes and the extensive SHM observed in this species, some reads could not be confidently assigned to a germline reference gene and were therefore classified as ‘Not assigned’. Other genes were labelled as ‘Ambiguous’ when multiple gene assignments were equally probable.

Nucleic acid sequences were translated into amino acids using the translate6frame tool from BBMAP 38.18 [46], and only sequences without stop codons were retained. Antibody FRs and CDRs were identified and confirmed using the species-agnostic IgMAT tool [31], which relies on consensus hidden Markov models built from multi-species reference datasets. Due to the limited diversity and relatively short porcine CDR3 regions, the CDR1, CDR2, and CDR3 sequences were concatenated to provide a longer and more informative sequence for downstream analysis. A cluster is defined as a group of protein sequences (BCR in this case) that share sequence similarity above a defined threshold, indicative of potential evolutionary or functional relatedness. Clustering was therefore carried out to group concatenated CDRs by sequence identity. USEARCH 11.0.667 [47] was used with a 96% similarity threshold. To account for variability in read depth across samples, cluster abundances were normalized to a 50 k read baseline. Accordingly, all data are presented here as relative read counts.

### BCR heavy-chain cluster selection and characterization

Clusters matching vaccination and/or infection kinetics were then selected (Table 1). First, for each cluster, the contribution of each time point was calculated as the proportion of reads at that time point relative to the total number of reads in the cluster, expressed as a percentage. A cluster was considered to follow a kinetic profile of interest when the meaningful time points post-vaccination and/or challenge contributed at least 10% of the cluster's total reads, while other time points contributed less than 10%. Day 0 was used as a negative control, as pigs were not yet immunized against PRV at this time, thus any clusters exceeding 10% at this time point were discarded. The 10% threshold was chosen empirically as it reliably captured biologically meaningful variations while limiting noise from low-frequency clusters.

**Table 1:** Clusters selection criteria.

Group	Day 0^a^	Post prime-vaccination^b^	Post boost-vaccination^c^	Post first challenge^d^	Post second challenge^e^
V: clusters exclusively observed after vaccination	−	**+/**−	**+**	−	−
C: clusters exclusively observed after challenge	−	−	−	**+/**−	**+**
VC: clusters observed after vaccination and challenge	−	**+/**−	**+**	**+/**−	**+**
U: clusters unrelated to vaccination and challenge kinetics or ambiguous	**Any other profile**				

^a^Presence or absence at day 0 with a threshold of 10% of the cluster's reads.

^b^Presence or absence at days 7, 14 or 20 with a threshold of 10% of the clusters’ reads.

^c^Presence or absence at days 25 or 28 with a threshold of 10% of the clusters’ reads.

^d^Presence or absence at days 40 or 42 with a threshold of 10% of the clusters’ reads.

^e^Presence or absence at day 64 with a threshold of 10% of the clusters’ reads.

Time points were assigned to four categories: post-prime vaccination (V1: days 7, 14, and 20); post-boost vaccination (V2: days 25 and 28); post-first challenge (C1: days 40 and 42), and post-second challenge (C2: day 64). Days 35, 49, 56, and 70 were considered too distant from vaccination or challenge to allow a confident identification of PRV-specific B cells. Only clusters appearing in at least two of these categories were retained, as recurrence was considered a necessary condition for a cluster to be considered biologically relevant with higher confidence, other clusters were arbitrarily considered as PRV-unrelated (U category). Lastly, we recorded the number of animals contributing to each cluster, which enabled the classification of clusters as private (found in one animal) or public (shared by two or more animals).

For each time point, selected clusters were quantified relative to the total number of clusters. Total relative read counts for each time point in selected clusters were added and divided by the total relative read count of the entirety of clustered reads.

### Statistical analysis

The proportion of animal counts in clusters was compared between selected and U clusters, with animal counts between 1 and 12. Per-category comparisons were conducted independently for each animal count using two-sample tests for equality of proportions. Considering heterogeneity in number of clusters in each category, Fisher's exact test was applied when expected counts were below five; otherwise, a chi-squared (χ^2^) test with Yates’ continuity correction was used (see Supplementary Table S3). Resulting *P*-values were adjusted for multiple testing using the Benjamini–Hochberg procedure. Statistical significance was reported using adjusted *P*-values.

Isotype distributions were compared between cluster profiles using a χ^2^ test of homogeneity. For each profile, the distribution of IGHM, IGHA, and IGHG genes was compared to the distribution of constant chains in *U* clusters. *P*-values were adjusted for multiple comparisons using the Benjamini-Hochberg procedure. Standardized residuals from the χ^2^ tests were examined to identify isotypes contributing to significant differences (see Supplementary Table S4).

## Results

### Clinical monitoring and anti-gB and anti-gE antibody detection after PRV vaccination and challenge

**Figure 2:**
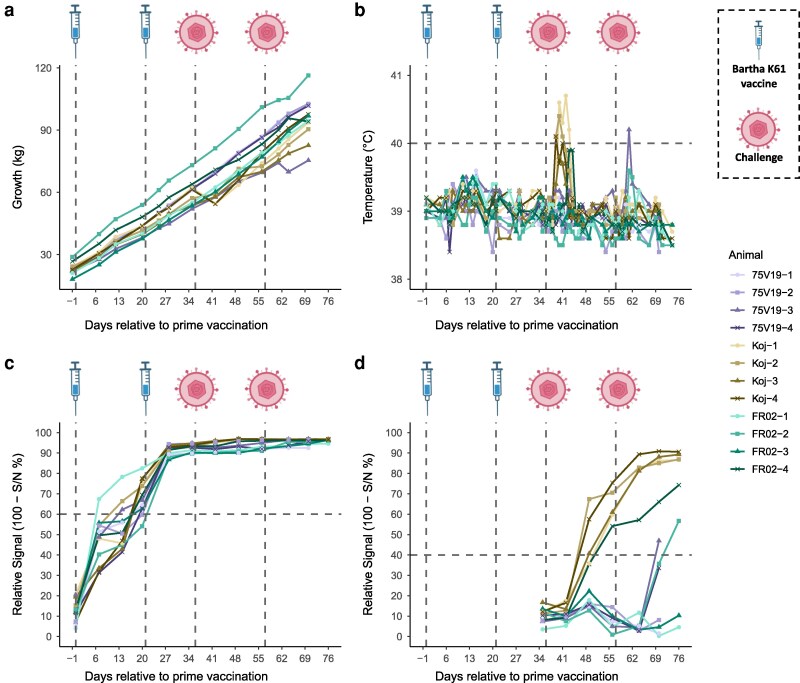
Serological responses and clinical parameters following vaccination and challenge with PRV. (a) Body weight (kg) and (b) rectal temperature (°C) were monitored longitudinally throughout the study. Dashed horizontal line in (b) indicates the hyperthermia threshold at 40°C. Competition ELISAs were performed to detect circulating antibodies against PRV glycoprotein gB (c) and gE (d). Results are expressed as relative signal (100–S/N%). According to the manufacturer's recommendations, positivity thresholds are <40% for gB and <60% for gE (dashed horizontal lines in panels C and D).

Following the two vaccinations, all the pigs continued to grow normally and gained approximately 1 kg per day (Fig. 2a). They also showed no increase in their body temperature (Fig. 2b). All pigs started to develop anti-gB antibodies from days 6 to 20 following the first vaccination. The levels of these antibodies remained high in the blood until the end of the experiment (Fig. 2c). As expected, no anti-gE antibodies were detected following the vaccinations (Fig. 2d). Following the first challenge, a transient period of rectal temperature above 40°C (1–3 days) was observed for all pigs in the Kojnok group but pig Koj-3, which had a 39.8°C rectal temperature after the first challenge (Fig. 2b). This hyperthermia was associated with a transient body weight loss of approximately 12% for only Koj-1 and Koj-4 pigs (Fig. 2a). Anti-gE antibodies were detected in the blood of the 4 pigs of this Kojnok group after the first challenge and remained at high levels until the end of the experiment. Following the challenge, transient increases in body temperature were observed in pigs FR02-2 and FR02-4 (up to 39.6°C and 39.9°C, respectively; Fig. 2b), with a brief and minor weight loss (1.7%) detected only in FR02-4 (Fig. 2a). Anti-gE antibodies were detected exclusively in these two pigs, appearing after the first and second FR02 challenge, respectively (Fig. 2d). In the 75V19 challenge group, only pig 75V19-3 showed clinical signs, including transient fever and a 5.8% weight loss following the second challenge (Figs 2a and b), together with detectable anti-gE antibodies from day 70 (Fig. 2d). No clinical signs or anti-gE antibodies were detected in the remaining pigs. Overall, all pigs mounted a robust and long-term anti-gB response after vaccination, while seven out of twelve animals (58%) developed an anti-gE response post-challenge. The presence of PRV was tested by qPCR in all pigs on day 76 in the tonsil and trigeminal ganglia, and the virus was only detected in the tonsils of all Kojnok-infected pigs and pig 75V19-3 (see Supplementary Table S2). Altogether, these observations indicate a close association between the occurrence of clinical signs, anti-gE seroconversion, and productive PRV infection following challenge, which was restricted to a subset of animals despite identical vaccination.

### Next-generation sequencing and quality control

Following sequence acquisition, 92.5% of samples achieved a Q30 quality score for at least 75% of their reads; low-quality reads were then removed using TrimGalore. Moreover, we explored whether samples had a constant number of reads in both runs (see Supplementary Figure S2a). Some samples exhibit under- or over-representation, especially the 56-day and 64-day samples of the first run. This bias would later be corrected by the normalization strategy we applied. We also explored the balance in isotype read counts as shown in Supplementary Figure S2b. As shown in Supplementary Figure S2c, the combined proportions of run 1 and run 2 isotypes were 33.8%, 29.5%, and 36.7% for IgG, IgM, and IgA, respectively.

### Selection of B-cell receptor heavy chains clusters consistent with PRV vaccination and/or infection kinetics

Biologically, B-cell activation in response to PRV is expected to result in B-cell expansion as measured by the frequency of antibody sequences in synchrony with vaccination and/or challenge events, consistent with the kinetics of an antigen-driven B-cell response [24]. To determine which heavy-chain clusters were potentially induced by PRV vaccination or challenge, we analysed the temporal dynamics of all BCR heavy-chain clusters and compared their respective kinetics with the schedule of the animal study. Among the 9 689 498 processed heavy-chain reads, 5 700 412 (58.8%) singletons were excluded for the downstream analysis, as they appeared only once in the dataset, and therefore could not be used for kinetic inference. The remaining 3 989 086 reads (41.2% of total reads) were distributed across 679 453 clusters. In addition, 45 870 clusters containing more than 10% of their normalized reads at day 0 (before immunization) were excluded as these clusters could not be related to PRV exposure. We defined eight cluster kinetic profiles compatible with the PRV exposure schedule, that can be grouped in three broad categories: Vaccination-associated (V) clusters that were strictly detected after the first and second vaccination or at least after the booster dose (called V1-V2 and V2 clusters, respectively), Challenge-associated (C) clusters that were strictly detected after the first and second challenge or at least after the second challenge (called C1-C2 and C2 clusters, respectively) and VC-clusters that were detected both after vaccination and challenge (V1-V2-C1-C2, V2-C1-C2, V1-V2-C2 and V2-C2 clusters). All the other clusters not following any of these profiles were considered as unlikely to be related to PRV treatments and were therefore classified as PRV-unrelated (U category).

Applying these criteria yielded 195 810 candidate clusters (the 20 most abundant clusters from each cluster kinetic profiles are illustrated in Supplementary Figure S3). As shown in Table 2, except for the V2 and C2 profiles, the selected clusters showed a clear predominance of clusters shared between at least two animals, namely public clusters. Notably, we selected 867 C1-C2 clusters of which 397 (45.8%) were exclusive to animals that displayed positive gE ELISAs results. In contrast, V2 and C2 clusters showed single, non-recurrent expansion peaks; therefore, subsequent analyses focused on clusters detected at multiple time points, which should be more likely to reflect PRV-associated, antigen-driven B-cell responses. Therefore, V2 and C2 clusters were excluded from subsequent analyses of selected clusters, leaving 10 499 selected clusters for downstream analysis. Altogether, this approach resulted in a curated set of recurrent heavy-chain clusters temporally associated with vaccination and/or challenge, which were subsequently analysed for their animal sharing patterns, isotype composition and relative abundance.

**Table 2:** Number and distribution of heavy-chain BCR clusters across kinetic profiles, classified as public or private.

Cluster profiles	Total number of clusters	Public clusters^a^	Private clusters
Total clusters	**697 453**	**105 324** (**15.1%)**	**592 129** (**84.9%)**
Selected clusters	**195 810**	**18 951 (9.7%)**	**176 859 (90.3%)**
**V**	**130 337**	**16 925 (13.0%)**	**113 412 (87.0%)**
V1-V2	8 671	7 725 (89.1%)	946 (10.9%)
V2	121 666	9 200 (7.6%)	112 466 (92.4%)
**C**	**64 512**	**1 245 (1.9%)**	**63 267 (98.1%)**
C1-C2	867	627 (72.3%)	240 (27.7%)
C2	63 645	618 (1.0%)	63 027 (99.0%)
**VC**	**961**	**781 (81.3%)**	**180 (18.7%)**
V1-V2-C1-C2	20	20 (100%)	0 (0%)
V2-C1-C2	95	84 (88.4%)	11 (11.6%)
V1-V2-C2	110	101 (91.8%)	9 (8.2%)
V2-C2	736	576 (78.3%)	160 (21.7%)
**U**	**501 643**	**86 373 (17.2%)**	**415 270 (82.8%)**

^a^Public clusters are defined as clusters detected in at least two animals; private clusters are detected in a single animal. Percentages are calculated relative to the total number of clusters within each row.

Of note, V(D)J gene usage remained constant in total and selected clusters across animals and time points as described in Supplementary Figure S4.

### Isotype expression is different between selected and PRV-unrelated clusters

As illustrated in Supplementary Figure S5, we compared isotype usage between PRV-unrelated (U) clusters and the different selected cluster profiles. Overall, selected clusters showed a statistically significant (*P* < 0.001) enrichment in IGHA expression compared with U clusters (Supplementary Table S3). In particular, IgA represented 52.16% of sequences in V1-V2 clusters and 68.97% in V1-V2-C1-C2 clusters, compared with 41.78% in U clusters.

In contrast, IGHG expression was reduced in several selected profiles. Notably, V2-C2 clusters exhibited lower IGHG usage (19.94%) compared with U clusters (33.14%). Similarly, IGHM expression was decreased in selected clusters, with V1-V2-C1-C2 clusters showing 11.09% IGHM compared with 25.08% in U clusters.

### Selected heavy-chain clusters display higher public cluster proportions compared to PRV-unrelated clusters

**Figure 3:**
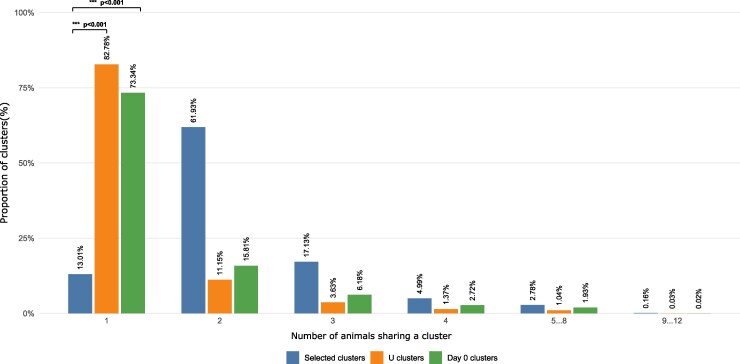
Distribution of the number of animals per cluster in selected, PRV-unrelated (U) clusters, and day 0 clusters. Following cluster selection, the number of animals contributing to each cluster was quantified. The proportion of clusters shared by one or more animals was calculated for selected clusters (excluding V2 and C2 profiles), and was compared to PRV-unrelated (U) clusters and day 0 clusters. Selected clusters (*N* = 10 499) showed a marked depletion of private clusters and a strong enrichment in public clusters compared to U clusters (*N* = 501 643) and day 0 clusters (*N* = 45 870). Statistical significance was assessed independently for each animal-count category using χ^2^ tests or Fisher’s exact tests where appropriate, with *P*-values adjusted for multiple comparisons. *** *P* < 0.001.

Following cluster selection, we quantified the number of animals encompassed in each cluster and compared these distributions between selected clusters (V, C, and VC), clusters with more than 10% reads to total clusters reads at day 0 (clusters that were excluded and used as negative control), and the PRV-unrelated clusters (U clusters). As shown in Fig. 3, the selected clusters consisted of almost 87% of public clusters, as opposed to only 17% of the U clusters or 27% for day 0 clusters (*P* < 0.001). For instance, 0.19‰ of the selected clusters were shared across 12 animals, compared with only 0.003‰ of U clusters (63-fold difference), and day 0 clusters did not comprise any cluster shared across 12 animals (*P* < 0.001). Altogether, these significant differences (Supplementary Table S4) indicate that clusters whose kinetics are consistent with PRV vaccination and/or challenge are markedly enriched in public clusters, in contrast to clusters classified as PRV-unrelated.

### The most frequent public clusters are shared across multiple treatment groups

**Figure 4:**
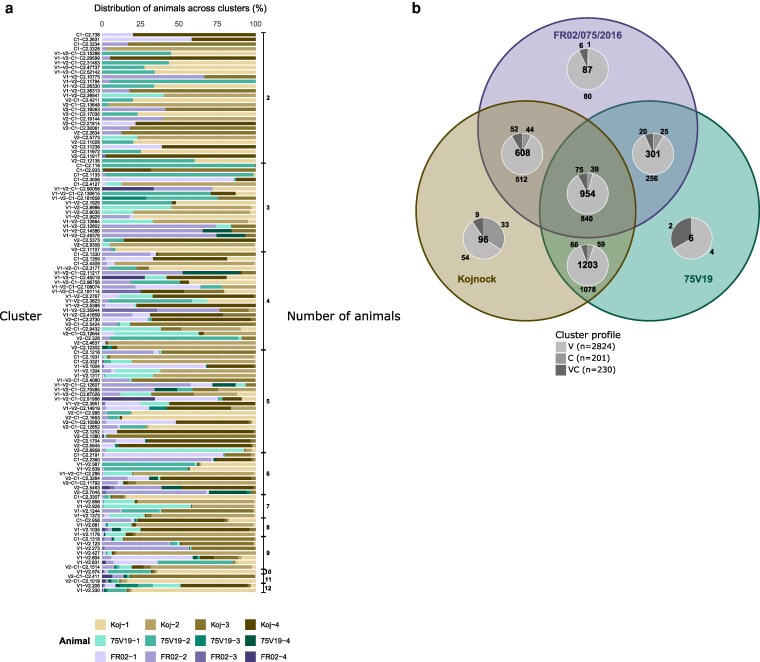
Inter-individual and inter-group sharing of selected public heavy-chain clusters. (a) Relative contribution of each of the twelve animals to 160 representative selected clusters. Clusters are ordered from top to bottom according to the number of contributing animals, as indicated on the right. Colours denote individual animals. Clusters detected in more than one animal are defined as public clusters. This panel illustrates the extent of inter-individual sharing among selected clusters. (b) Venn diagram showing the distribution of public heavy-chain clusters (≥10 normalized reads) across the three challenge groups (Kojnok, 75V19, and FR02/075/2016). All pigs received the same Bartha K61 vaccine and differed only by the challenge strain. Numbers indicate the total clusters in each intersection. Pie charts represent the proportion of vaccination-associated (V), challenge-associated (c) and vaccination-and-challenge-associated (VC) kinetic profiles within each group. The extensive overlap across groups highlights a high degree of shared, vaccine- and/or PRV-associated responses.

To ensure that downstream comparisons were based on reliably detected sequences, we restricted the analysis to public clusters represented by at least 10 normalized reads; the remaining clusters retained 72% of the selected clusters normalized reads. Because four pigs were sampled only at days 25 and 40, clusters shared by more than eight animals are expected to be underrepresented, as limited sampling reduces the likelihood of detecting clusters present in all individuals. Consistently, clusters shared by two to eight animals accounted for 99.48% of the public-selected clusters, leaving only 17 clusters (5.2‰) shared by nine to twelve animals. Among the 17 clusters shared by nine to twelve animals, 13 (76.5%) belonged to the V1–V2 kinetic profile, three (17.6%) to the V2-C1-C2 profile, and one (5.9%) to the C1–C2 profile. Notably, two V1–V2 clusters were detected in all 12 animals. For visual clarity, the between-animals distributions of the 20 most abundant public clusters from each kinetic profile are presented in Fig. 4a.

In addition to inter-individual sharing, a large proportion of public clusters were also shared between pigs challenged with different PRV-strains. As shown in Fig. 4b, among the 3 255 public clusters with 10 or more normalized reads, only 189 (5.81%) were exclusive to a single challenge group, whereas 954 clusters (29.31%) were shared across all three challenge groups. These widely shared clusters likely reflect recognition of epitopes shared between the Bartha K61 vaccine strain and/or the different challenge strains. Focusing on VC clusters, 75 of the 230 clusters (32.6%) were shared by all three challenge groups, suggesting that these responses target epitopes conserved between the vaccine strain and all three challenge strains. A further 66 VC clusters (28.7%) were shared only between pigs challenged with Kojnok and 75V19, 52 (22.6%) only between the Kojnok and FR02 groups, and 20 (8.7%) only between 75V19 and FR02 groups. Thus, while many VC clusters likely recognize conserved PRV antigens, others may correspond to antibodies targeting epitopes shared only between the vaccine strain and a subset of the challenge strains.

Altogether, these findings highlight a strong predominance of public clusters, characterized by extensive sharing between animals and across challenge groups, and a subset of clusters showing particularly high levels of convergence.

### Cluster quantification leads to the identification of key sampling time points

**Figure 5:**
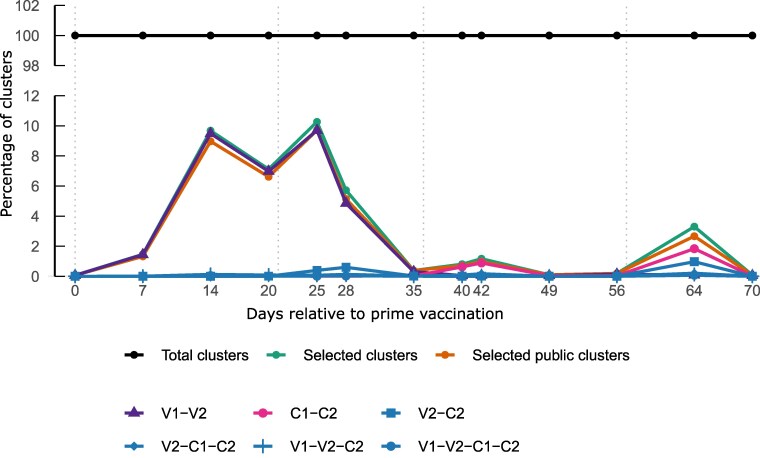
Temporal quantification of selected and public selected BCR heavy-chain clusters. Relative contribution (%) of selected clusters and selected public clusters to the total normalized read counts across all sampling time points. Total clusters represent 100% of reads at each time point and are shown as a reference baseline. Curves correspond to all selected clusters, selected public clusters, and individual kinetic profiles. Vertical dashed lines indicate vaccination and challenge time points. Selected clusters peaked after booster vaccination (day 25), indicating maximal enrichment of candidate PRV-specific B cells, while challenges induced smaller and more transient increases. This relative quantification highlights key time points for targeted sampling of PRV-reactive B-cell populations.

To estimate the relative abundance of candidate PRV-specific antibody clusters throughout the study, we compared the normalized number of reads in total clusters, selected clusters, and public selected clusters (Fig. 5). Vaccination induced a marked increase in the proportion of likely PRV-specific B cells, with days 14, 20, and 25 showing the highest enrichment, reaching up to 10.26% of the humoral response at day 25. Although representing a smaller proportion of the total B-cell repertoire, both challenge infections also elicited an increase in PRV-specific candidate clusters (Fig. 5). This relative quantification provides an overview of the temporal evolution of selected B-cell populations putatively induced by vaccination and/or challenge and highlights the first contact with antigens as dominant drivers of detectable circulating BCR expansions in this experimental setting.

Altogether, these results demonstrate that kinetic clustering of BCR heavy-chain sequences enables the identification of candidate PRV-induced antibody populations and their temporal dynamics. The defined kinetic profiles, the restricted expansion peaks following vaccination or challenge, and the high degree of sharing between animals and across challenge strains, collectively indicate a strongly convergent humoral response. These clusters therefore represent plausible PRV-specific or PRV-associated antibody lineages. Finally, their quantitative enrichment pinpoints key sampling time points, particularly following the booster vaccination, which may guide future single-cell studies aimed at recovering paired heavy and light chains for functional validation.

## Discussion

PRV infection induces strong humoral and cellular immune responses directed primarily against glycoproteins gB, gC, gD, and gE [48, 49]. Live or inactivated attenuated gE-deleted vaccines such as Bartha K61 are widely used, as they provide effective clinical protection against classical PRV strain while enabling a DIVA strategy [48]. In agreement with this, all pigs in the present study developed robust anti-gB antibody responses following vaccination, while no anti-gE antibodies were detected prior to challenge, confirming the expected immunological response to the Bartha K61 strain. Clinical signs following challenge were mild and transient in seven animals, indicating vaccine-mediated protection [29]. However, the occurrence of clinical signs and gE seroconversion in a subset of animals is consistent with previous reports [50] showing reduced efficacy of Bartha K61 against genetically divergent PRV strains. Anti-gE antibodies arose exclusively in animals infected with wild-type PRV strains, notably in all Kojnok-challenged pigs and in a subset of FR02- and 75V19-challenged pigs. This serological pattern was consistent with partial infection, as PRV was detected in tonsils at necropsy in all Kojnok-challenged pigs and in pig 75V19-3. Altogether, these observations confirm successful induction of PRV-specific immunity and validate the experimental framework for exploring PRV-driven B-cell responses at the repertoire level against classical PRV strain.

To capture antigen-specific B cell responses, longitudinal PBMC sampling was performed at biologically relevant time points, close to the expected peaks of clonal expansion following vaccination and challenge. Heavy-chain BCR variable regions were amplified and sequenced using a NGS strategy, and antibody populations were analysed by clustering concatenated CDRH1-CDRH2-CDRH3 amino acid sequences. This approach was chosen to track the kinetics of BCR clusters and to identify those consistent with vaccination and/or challenge events, as expected for antigen-driven responses.

As a first filtering step, clusters detected at day 0, corresponding to the pre-immune baseline, were excluded to focus analyses on vaccine- or infection-associated sequences. This yielded 195 810 clusters with kinetics compatible with at least one immunization or challenge step. However, most of these clusters were detected at a single time point in a given animal. Because recurrence across multiple time points provides stronger evidence of clonal expansion and antigen-driven selection, only clusters detected at two or more relevant time points were retained for downstream analyses. This stringent filtering reduced the dataset to 10 499 clusters. Importantly, exclusion from this final set does not imply lack of biological relevance, as it cannot be excluded that antigen-specific BCRs can also be found among rare, transient clusters.

The retained clusters were classified into three kinetic categories: vaccination-associated (V), challenge-associated (C), and vaccination- and challenge-associated (VC). We hypothesized that V clusters correspond to antibody responses induced by Bartha K61 vaccination, C clusters to responses induced by challenge infection, including potential gE-specific clones, and VC clusters to vaccine-induced clones recalled or boosted upon challenge.

Strikingly, only 13% of these clusters were private, whereas the remaining 87% were shared by at least two animals, indicating a high degree of inter-individual convergence. Public clusters exhibited a broad range of sharing levels, from clusters detected in pairs of animals to clusters shared across all individuals. Notably, two V1-V2 clusters were detected in all twelve pigs, representing complete convergence within this cohort. Convergent BCR responses of more limited extent have previously been reported following PRV infection in mice [21], as well as in humans vaccinated against *Haemophilus influenzae* type b or meningococcal polysaccharides, and in responses to dengue, Zika, SARS-CoV-2, influenza and hepatitis C virus [25–27]. In this context, public clonotypes often correspond to antibodies targeting immunodominant or structurally conserved epitopes and are widely used as markers of antigen-specific immune responses. The extensive sharing observed in the present study therefore strongly suggests that many of the public clusters identified here are PRV-induced.

This interpretation is further supported by the observation that only a small fraction of public clusters were exclusive to a single PRV challenge strain. Instead, most public clusters were shared across challenge groups, reflecting recognition of epitopes common to both the Bartha K61 vaccine strain and the different pathogenic strains used for challenge. Such conserved epitopes likely reside on PRV glycoproteins known to harbour cross-reactive and neutralizing sites, such as gB and gD [48] (Supplementary Figure S1). Moreover, gD is known to induce protective immune responses [51]. In addition, seven animals developed anti-gE antibodies as detected by ELISA, and these animals were specifically represented in 397 C1-C2 clusters, whereas the remaining five animals were absent from these clusters. These C1-C2 clusters may therefore include gE-specific BCR heavy chains. Although the Kojnok strain used in this study yielded a weak positive signal for mycoplasma contamination (Ct 35.4), the extensive sharing of public clusters across all challenge groups argues that the dominant convergent responses identified here are driven by PRV antigens rather than by extraneous contaminants or adjuvant effects.

PRV is neurotropic but also replicates in the respiratory tract [48]. Accordingly, IgG is expected to play a major role in systemic vaccination response and antiviral immunity, while IgA response may be favoured following oronasal infection. In line with this, selected clusters showed an increased representation of IgA compared with PRV-unrelated clusters, consistent with class switching in response to vaccination and challenge and with mucosal involvement following oronasal infection. This shift was particularly marked in V1–V2 and V1–V2–C1–C2 groups, suggesting a potential role for IgA in both systemic and mucosal responses following vaccination and challenge. While serum IgA is predominantly monomeric and mucosal IgA is typically dimeric, the present data do not distinguish between these forms. Although a parallel increase in IgG might also have been expected, IgG representation was equivalent to or lower than that observed in *U* clusters, while IgM was globally reduced in selected clusters. These patterns likely reflect the complexity of isotype dynamics in bulk PBMC repertoires and the timing of sampling relative to class-switch events.

Quantification of the relative abundance of selected clusters across time points provided additional insight into the dynamics of the PRV-specific humoral response. Vaccination induced the highest enrichment of candidate PRV-specific heavy-chain clusters, peaking around day 25 post-prime-vaccination, when selected clusters accounted for up to 10% of the repertoire. Challenge also induced an increase in PRV-associated clusters, albeit of lower magnitude, likely reflecting more rapid viral clearance following priming. This reduced expansion after challenge is consistent with the presence of circulating vaccine-induced antibodies, which are expected to limit viral replication and antigen availability, thereby attenuating the magnitude of the secondary B-cell response [52]. While such relative quantification must be interpreted cautiously, as NGS read counts do not directly correspond to cell numbers, these analyses nonetheless identify sampling windows that are particularly enriched for PRV-reactive B cells and would therefore be well suited for downstream single-cell studies.

Several limitations of this study should be acknowledged. First, it captures circulating B-cells contributing to the antibody response, such as plasmablasts; it may overlook other relevant populations, especially plasma cells residing in lymph nodes or bone marrow. Second, the analysis was restricted to B-cell heavy-chain variable regions, whereas full antigen specificity depends on paired heavy and light chains. Third, bulk repertoire sequencing does not preserve native chain pairing and is subject to stochastic biases introduced during pre-NGS PCR amplification [41]. Fourth, the stringent cluster-selection criteria enrich for high-confidence antigen-associated clusters but necessarily exclude rare or unconventional lineages. Finally, the use of genetically similar, age-matched SPF pigs housed under identical conditions maximized the detectability of convergent responses [26], and may limit direct extrapolation to more heterogeneous pig populations.

Convergence in porcine BCR repertoires is further facilitated by intrinsic features of the pig immunoglobulin locus, including a reduced number of functional V, D, and J genes and dominant usage of the VH3 family [22, 53, 54]. This restricted combinatorial diversity increases the relative contribution of SHM to repertoire diversification [55]. SHM-driven convergence has been described in humans and other species [56], and similar patterns have been reported in pigs in response to foot-and-mouth disease virus, influenza virus, and other pathogens [55, 57]. The present study extends these observations by demonstrating widespread convergent BCR expansions within and across groups of PRV-vaccinated and challenged pigs, including two clusters detected in all individuals. To our knowledge, such a level of convergence has not previously been reported in large livestock animals. In conclusion, bulk heavy-chain repertoire analysis enabled the identification of thousands of putative vaccination- and/or challenge-associated BCR clusters, including numerous public clusters whose kinetics and inter-individual sharing strongly support PRV-induced origins. In the future, such studies on related species would be interesting, especially on Meishan pigs which are likely to be exposed to PRV infection and are known to have differences in their immune reactions compared to European pig breeds [58]. The use of SLA-defined pigs [59] would also help to reduce the possible variability of the immune response introduced by polymorphism in the SLA of pigs. These findings provide a robust framework for targeted recovery of full antibody sequences. Future work should focus on single-cell BCR sequencing of selected samples collected at key time points, enabling pairing of heavy and light chains and direct assessment of antigen specificity. Using those sequences, in silico modelling may allow for the prediction of BCR-antigen interactions, as already shown in humans. For example, a site near the apex of the prefusion conformation of respiratory syncytial virus fusion protein was identified as a potential vaccine antigen thanks to the paired-sequencing of antibody variable heavy (VH) and variable light (VL) encoded by memory B cells of healthy human donors [60]. Such approaches are increasingly needed, as the emergence of genetically and antigenically divergent PRV strains challenges the breadth of protection conferred by existing vaccines (including Bartha K61), especially in China, where those strains are circulating and their zoonotic potential raises concern [61]. This context highlights the importance of identifying conserved and cross-protective epitopes. This integrated strategy will allow validation of PRV-specific public antibodies, mapping of conserved epitopes, and deeper understanding of humoral response convergence following vaccination and infection in pigs.

## Supplementary Material

kyag009_Supplementary_Data

## Data Availability

For the purpose of Open Access, the author has applied a CC BY public copyright licence to any Author Accepted Manuscript (AAM) version arising from this submission. The data presented in this study are available as BioProject accession PRJNA1468510.

## Permission to reproduce

Figure 1 created in BioRender. Biorender, V. (2026) https://BioRender.com/yuah2lp.

## Abbreviations:

AID: activation-induced cytidine deaminase
BCR: B-cell receptor
CDR: Complementarity determining region
FR: Framework region
Ig: Immunoglobulin
IgMAT: Immunoglobulin Multi-species Annotation Tool
PBMC: Peripheral blood mononuclear cell
PR: Pseudorabies
PRV: Pseudorabies virus
SHM: Somatic hypermutation
SPF: Specific pathogen-free
