# Supplementary material for: Profiling of porcine B-cell receptor heavy-chain repertoires indicates the development of a wide public pseudorabies virus-specific immune response after vaccination and challenge
Source: Discov Immunol. 2026 May 5;5(1):kyag009. doi: 10.1093/discim/kyag009 (PMC13225268; doi:10.1093/discim/kyag009)
Supplement: kyag009_Supplementary_Data [file kyag009_supplementary_data.zip › FigS2.pdf]

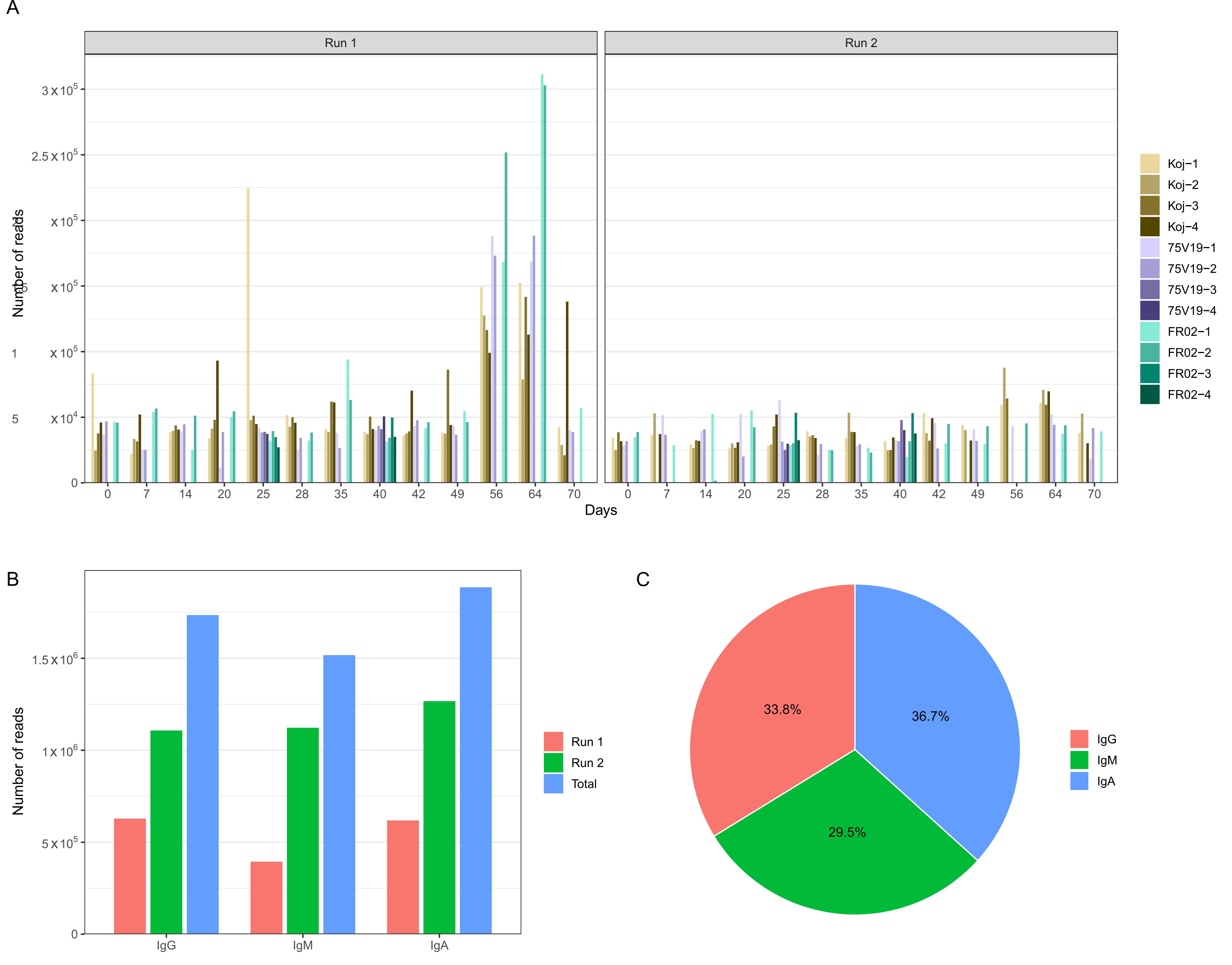

Figure S2. Sequencing quality control. (A) Following sequence acquisition, the number of reads obtained for each sample and for each sequencing run was assessed to ensure suitability for downstream analyses. (B) Read counts for each immunoglobulin isotype (IgG, IgM and IgA) were quantified separately for each sequencing run, as well as for the combined dataset (runs 1 and 2). (C) The relative proportions of each isotype within the total read pool were subsequently calculated.
