# Supplementary material for: Profiling of porcine B-cell receptor heavy-chain repertoires indicates the development of a wide public pseudorabies virus-specific immune response after vaccination and challenge
Source: Discov Immunol. 2026 May 5;5(1):kyag009. doi: 10.1093/discim/kyag009 (PMC13225268; doi:10.1093/discim/kyag009)
Supplement: kyag009_Supplementary_Data [file kyag009_supplementary_data.zip › FigS3.pdf]

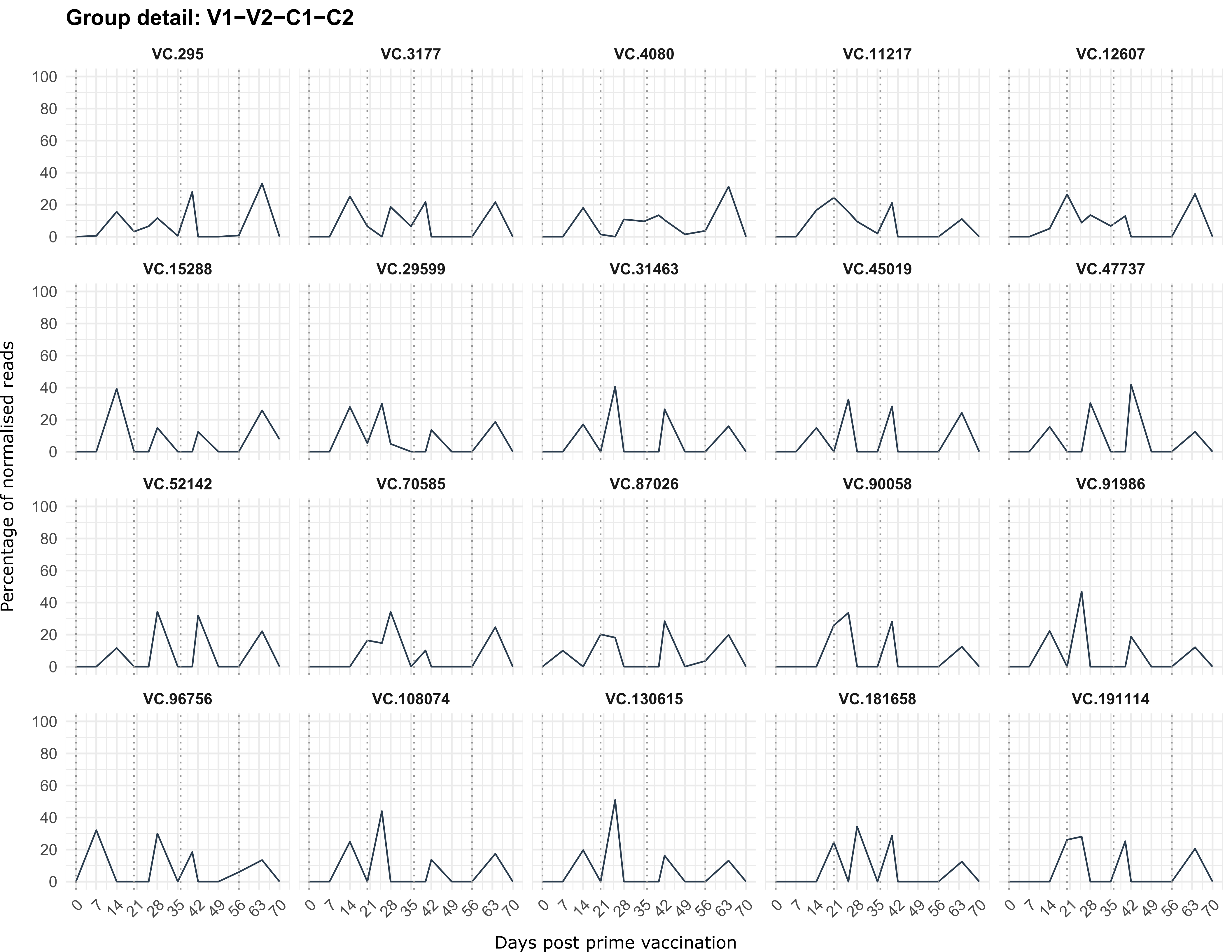

Group detail: V1−V2−C1−C2
VC.295
VC.3177
VC.4080
VC.11217
VC.12607
VC.15288
VC.29599
VC.31463
VC.45019
VC.47737
VC.52142
VC.70585
VC.87026
VC.90058
VC.91986
VC.96756
VC.108074
VC.130615
VC.181658
VC.191114
Percentage of normalised reads
0
20
40
60
80
100
0 7 14 21 28 35 42 49 56 63 70
Days post prime vaccination

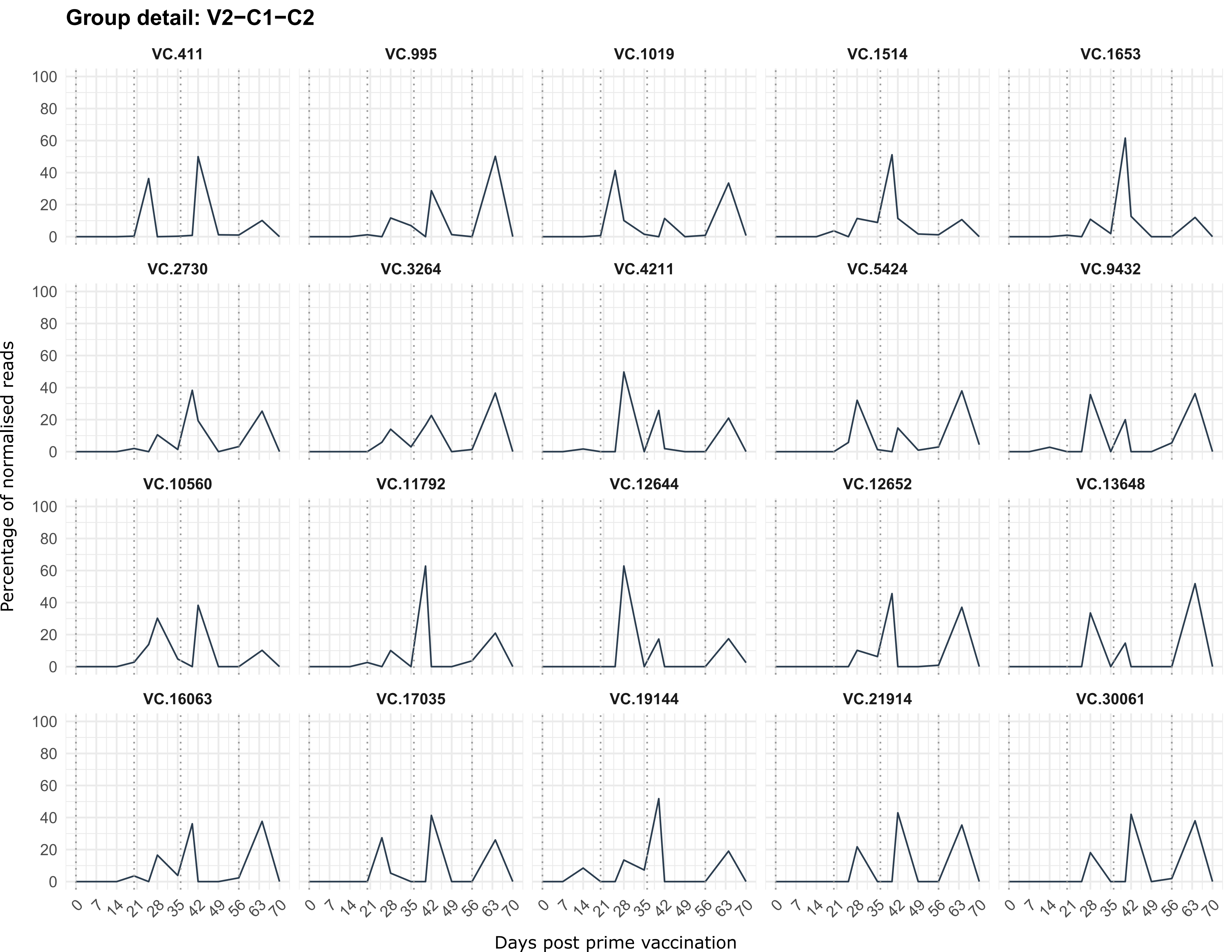

Group detail: V2−C1−C2
VC.411
VC.995
VC.1019
VC.1514
VC.1653
VC.2730
VC.3264
VC.4211
VC.5424
VC.9432
VC.10560
VC.11792
VC.12644
VC.12652
VC.13648
VC.16063
VC.17035
VC.19144
VC.21914
VC.30061
Percentage of normalised reads
0
20
40
60
80
100
0 7 14 21 28 35 42 49 56 63 70
Days post prime vaccination

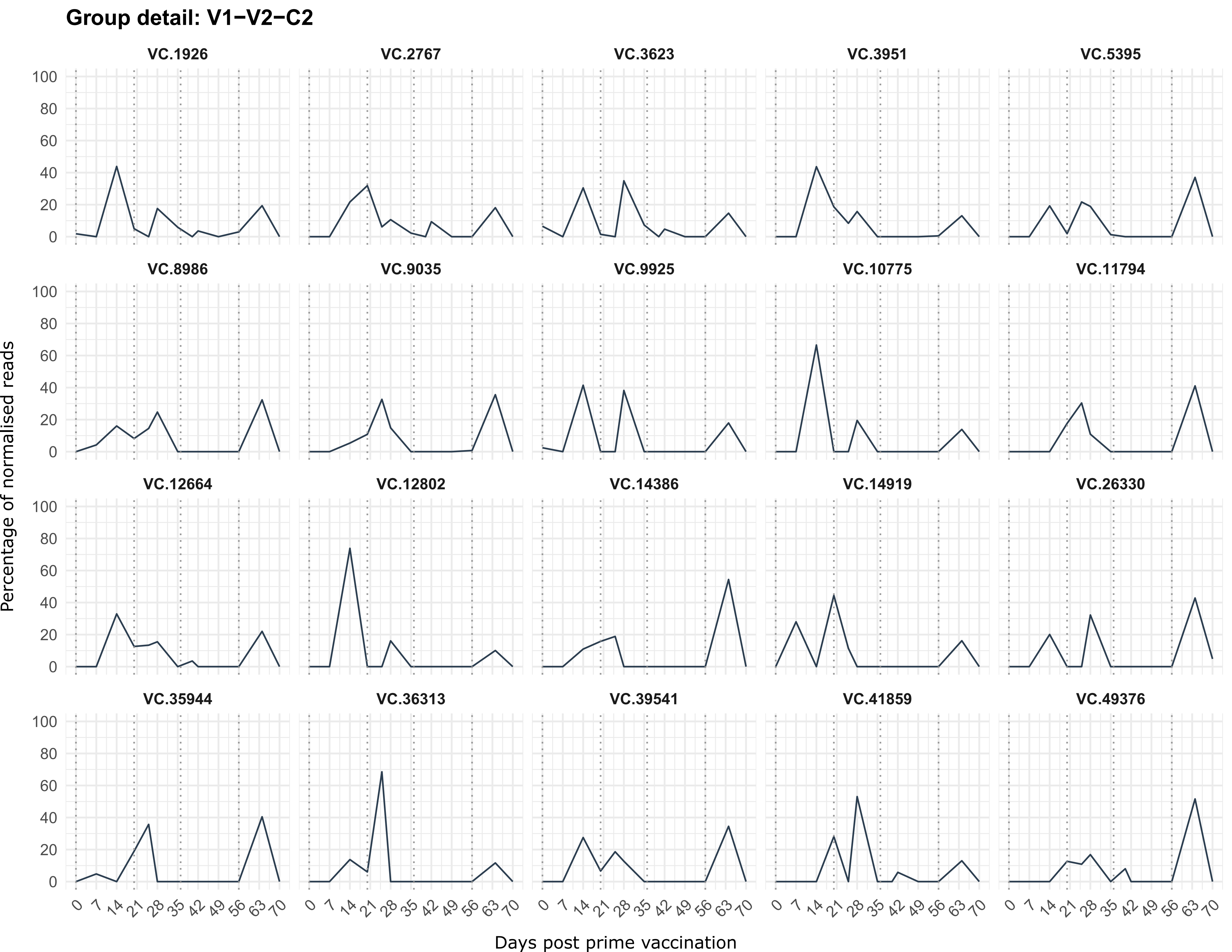

Group detail: V1−V2−C2
VC.1926
VC.2767
VC.3623
VC.3951
VC.5395
VC.8986
VC.9035
VC.9925
VC.10775
VC.11794
VC.12664
VC.12802
VC.14386
VC.14919
VC.26330
VC.35944
VC.36313
VC.39541
VC.41859
VC.49376
Percentage of normalised reads
0
20
40
60
80
100
0 7 14 21 28 35 42 49 56 63 70
Days post prime vaccination

Group detail: V2−C2

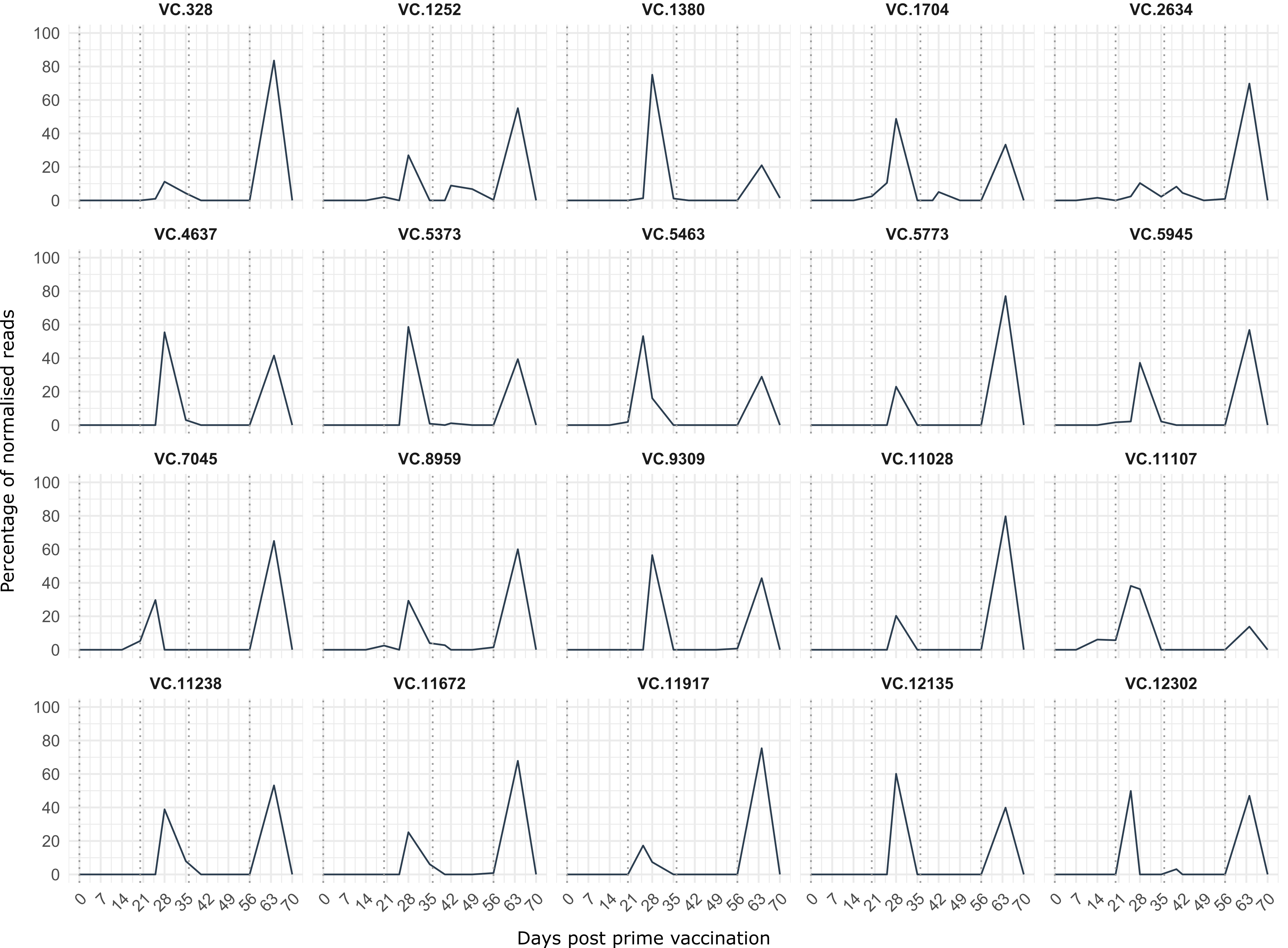

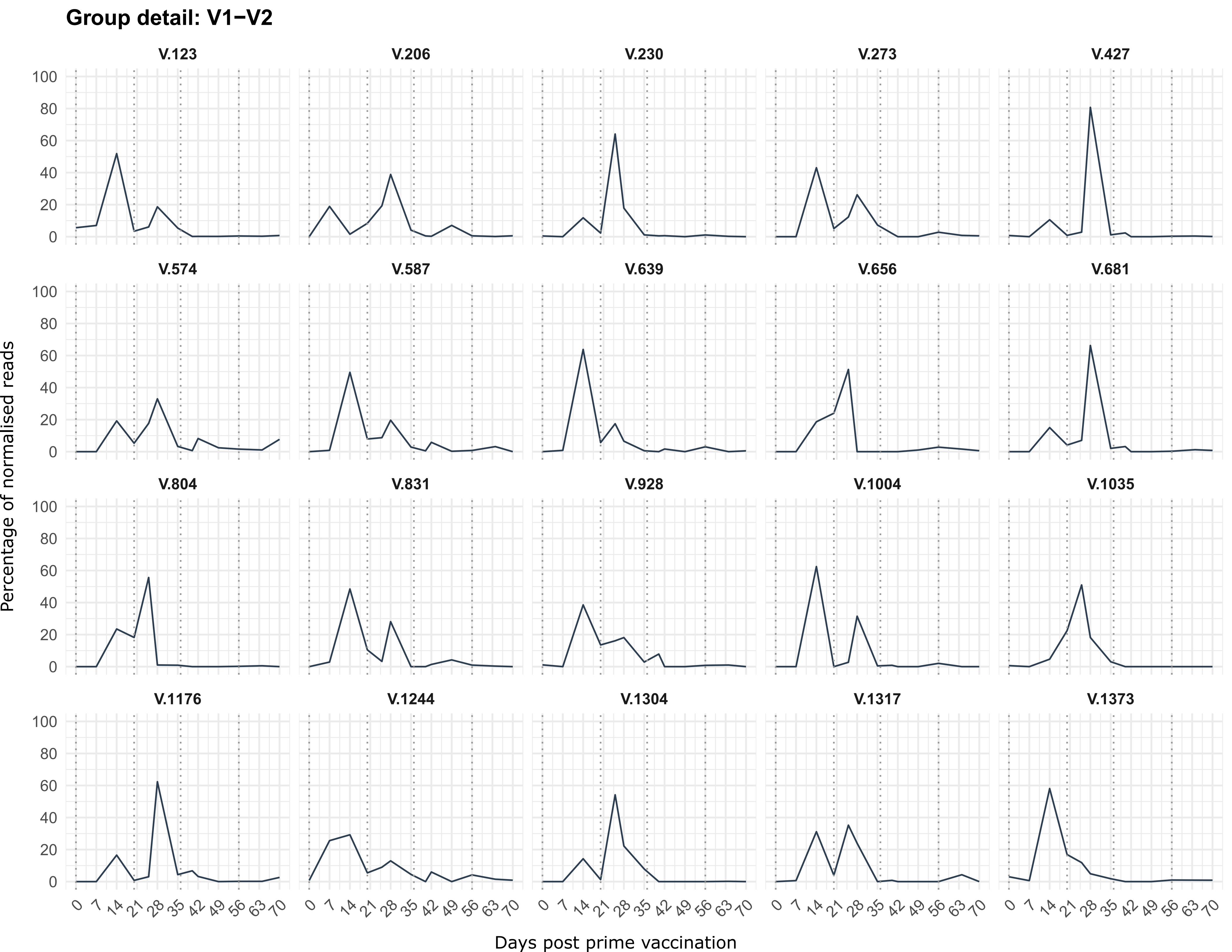

Group detail: V1−V2
V.123
V.206
V.230
V.273
V.427
V.574
V.587
V.639
V.656
V.681
V.804
V.831
V.928
V.1004
V.1035
V.1176
V.1244
V.1304
V.1317
V.1373
Percentage of normalised reads
0
20
40
60
80
100
0 7 14 21 28 35 42 49 56 63 70
Days post prime vaccination

Group detail: V2

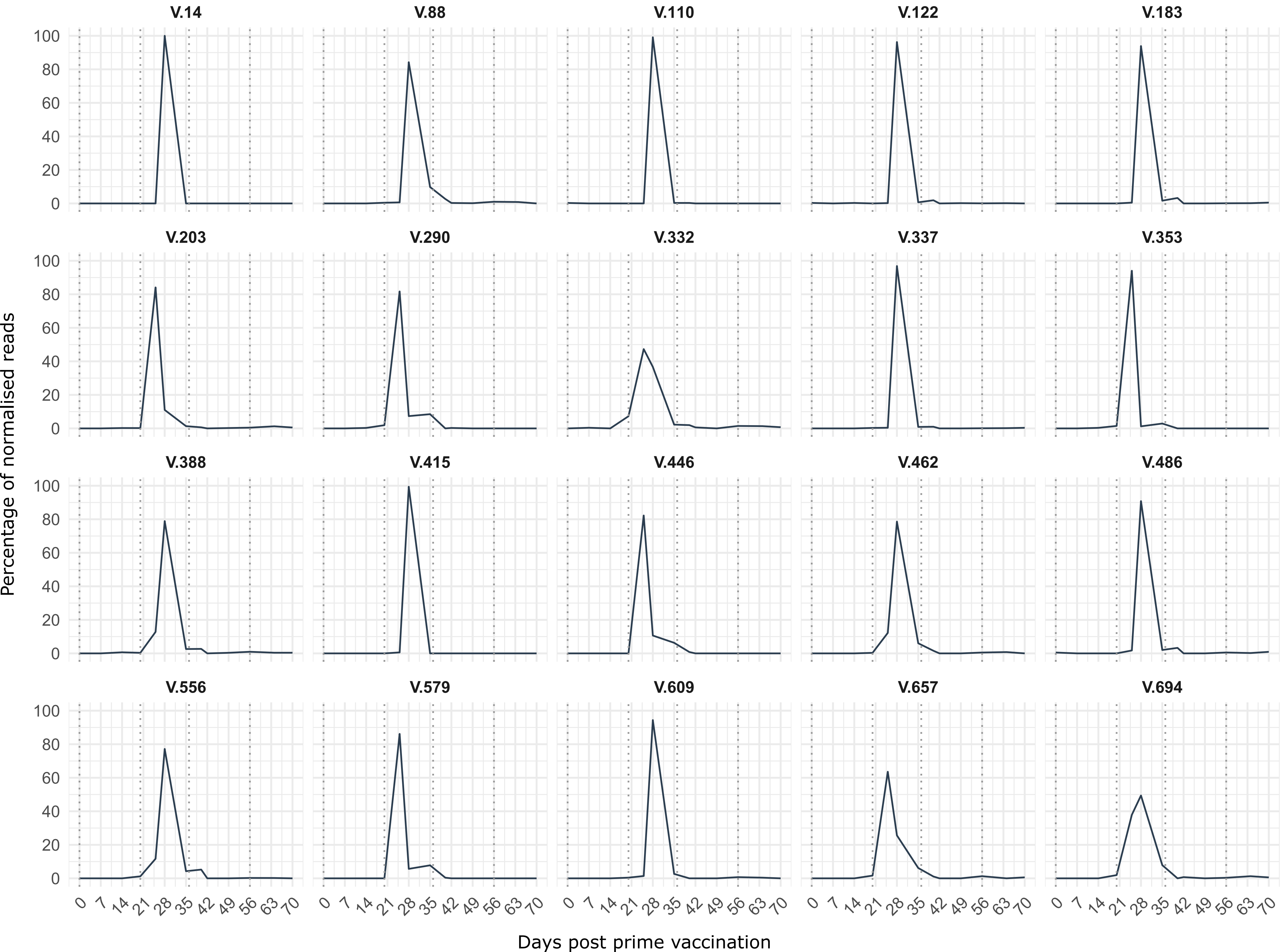

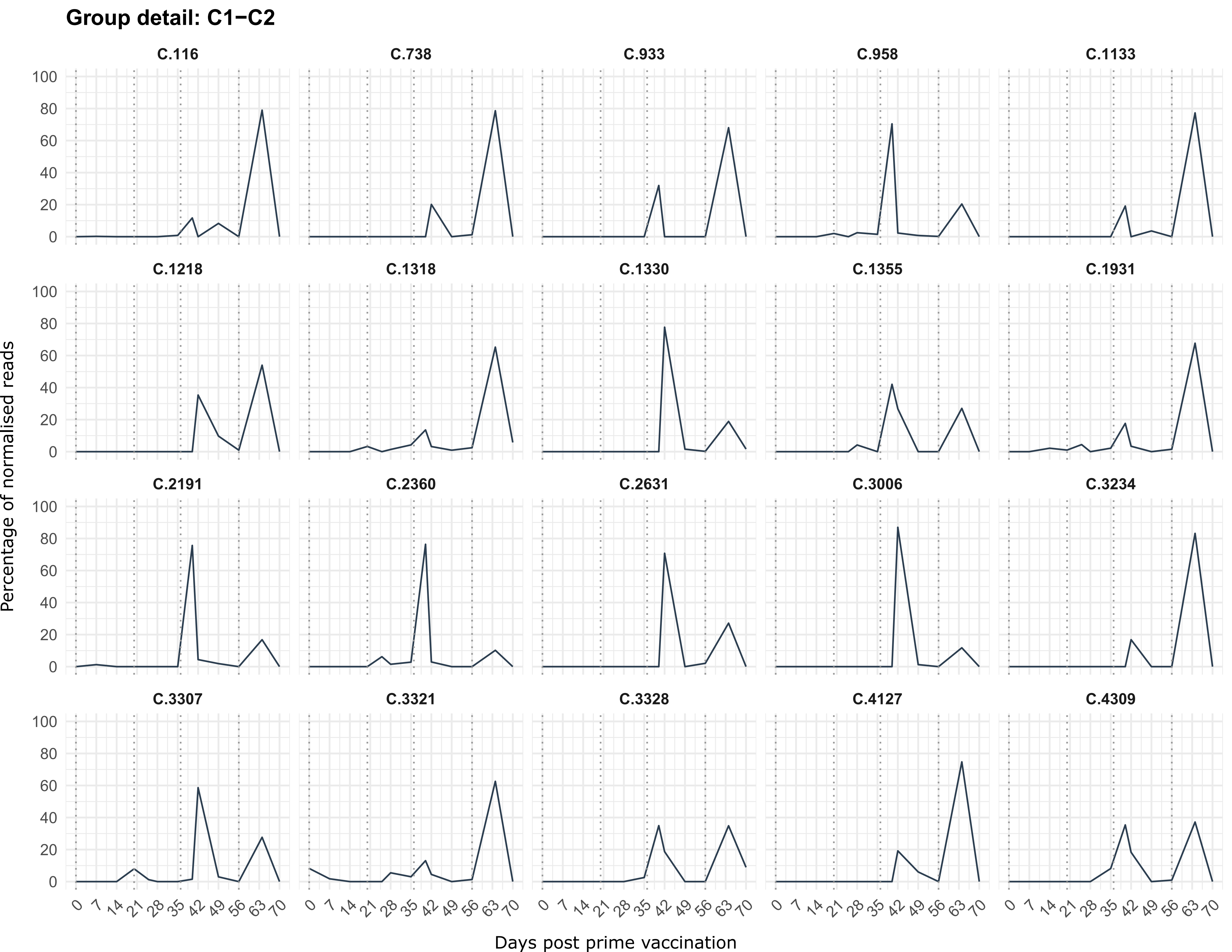
Group detail: C1−C2
C.116
C.738
C.933
C.958
C.1133
C.1218
C.1318
C.1330
C.1355
C.1931
C.2191
C.2360
C.2631
C.3006
C.3234
C.3307
C.3321
C.3328
C.4127
C.4309
Percentage of normalised reads
Days post prime vaccination
0
20
40
60
80
100
0
7
14
21
28
35
42
49
56
63
70

Group detail: C2

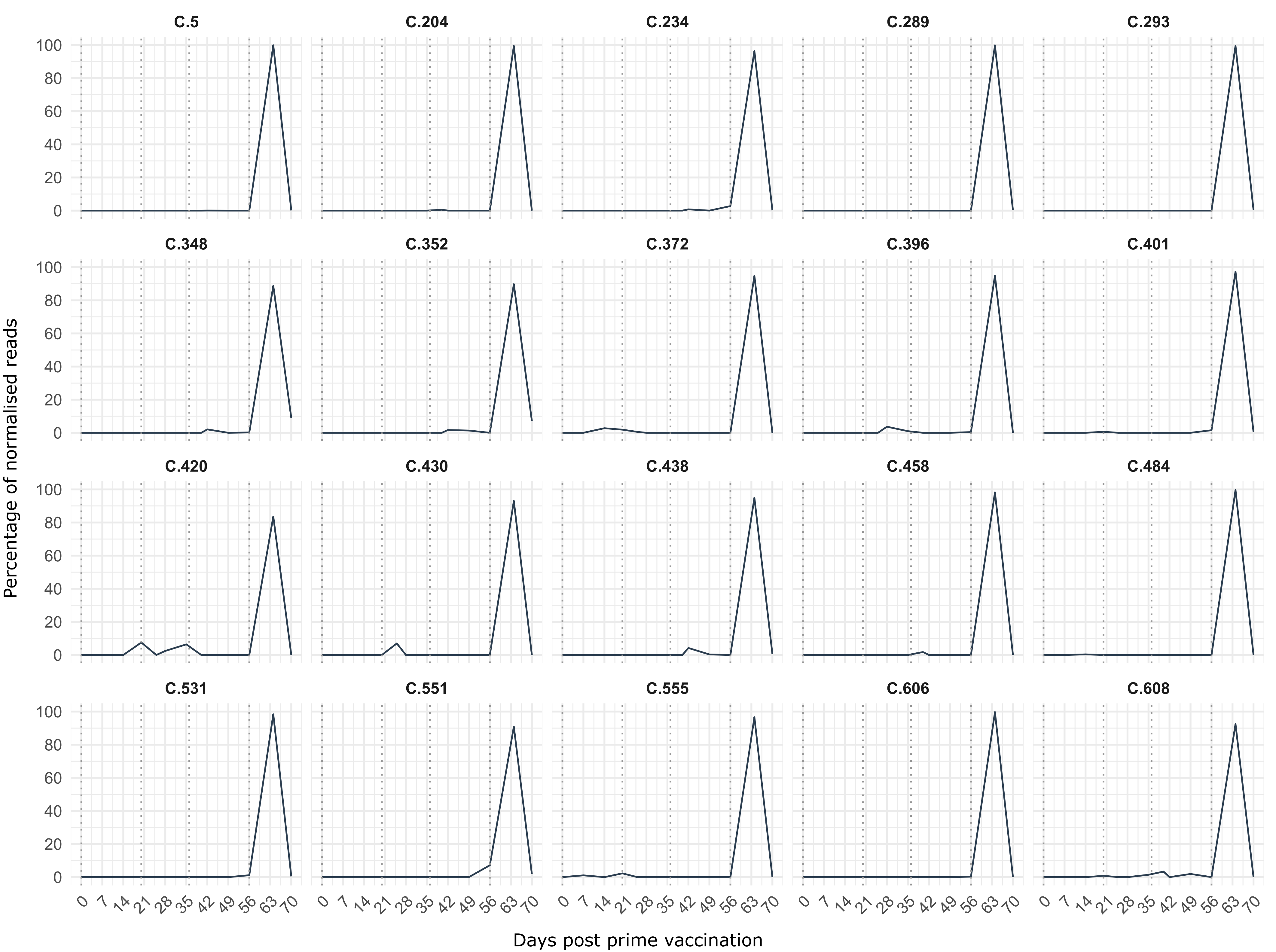

Figure S3. Kinetics of the 20 most abundant clusters for each kinetic profile. Temporal dynamics of the 20 most abundant heavy-chain clusters for each kinetic profile, defined by the occurrence of expansion peaks after prime vaccination, booster vaccination, first challenge, and/or second challenge. Each plot corresponds to one cluster and shows normalized read counts across longitudinal sampling time points
