# Supplementary material for: Profiling of porcine B-cell receptor heavy-chain repertoires indicates the development of a wide public pseudorabies virus-specific immune response after vaccination and challenge
Source: Discov Immunol. 2026 May 5;5(1):kyag009. doi: 10.1093/discim/kyag009 (PMC13225268; doi:10.1093/discim/kyag009)
Supplement: kyag009_Supplementary_Data [file kyag009_supplementary_data.zip › FigS4.pdf]

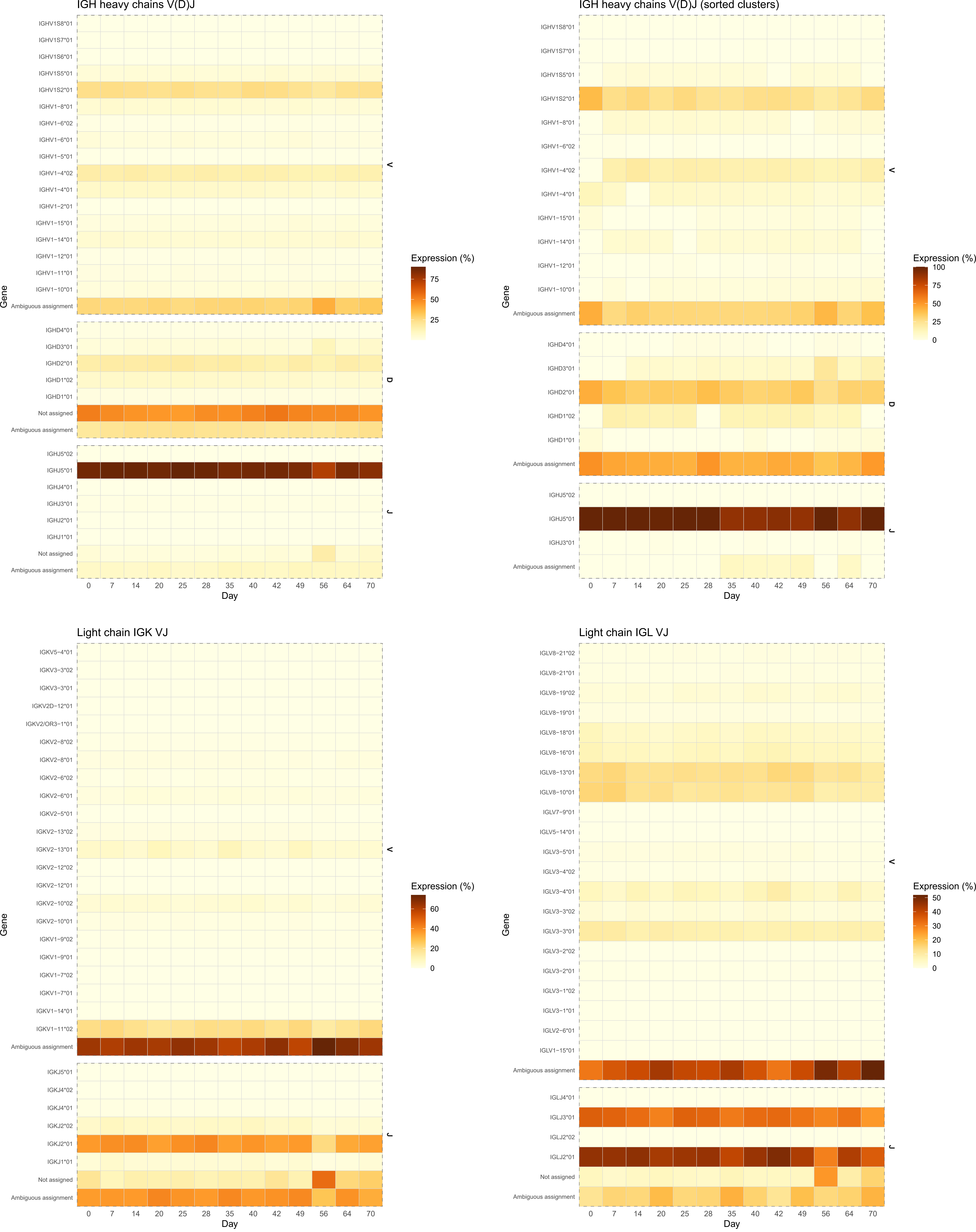

Figure S4. V(D)J gene usage is unchanged in selected clusters compared with the total antibody population. V(D)J gene usage was analysed in heavy- and light-chain sequences to assess whether selection of clusters based on vaccination and challenge kinetics was associated with shifts in gene segment usage (Figure S2; Table S2). Across both heavy and light chains, more than 25% of V(D)J assignments were ambiguous.

For heavy chains, VH gene usage was dominated by two genes, IGHV1S2*01 and IGHV1-4*02, which represented approximately 23.07% and 14.85% of all expressed VH genes, respectively, in both the total repertoire and the selected clusters. Other VH genes individually accounted for ~5% or less of VH usage, while 28.27% of VH assignments were ambiguous. DH gene usage was mainly represented by IGHD2*01, with minor contributions from IGHD1*02 and IGHD3*01, accounting for 16.15%, 7.01%, and 4.47% of identified DH genes, respectively. A large fraction of DH genes were either not assigned (48.60%) or ambiguously assigned (21.27%). JH gene usage was strongly dominated by IGHJ5*01 (85.97%), whereas IGHJ3*01 and IGHJ5*02 represented 0.75% and 0.20% of reads, respectively; the remaining assignments were not assigned (4.34%) or ambiguous (8.71%).

Light-chain VJ gene usage was also assessed. For lambda chains, IGLV8-13*01 and IGLV8-10*01 represented 13.42% and 12.28% of VL genes, respectively. Other identified IGLV genes (including IGLV8-1902, IGLV8-1801, IGLV8-1601, IGLV3-401, and IGLV3-3*01) each accounted for less than 10% of VL usage, while 40.23% of assignments were ambiguous. IGLJ usage was primarily distributed between IGLJ2*01 (42.90%) and IGLJ3*01 (32.08%), with minor contributions from IGLJ2*02 (0.10%) and IGLJ4*01 (0.04%); the remaining assignments were either not assigned (8.06%) or ambiguous (16.82%).

For kappa chains, VK usage was dominated by IGKV1-11*02 (19.02%), followed by IGKV2-13*01 (5.97%), IGKV2-10*02 (3.79%), and IGKV2-6*01 (2.08%). All other VK genes individually represented 1% or less of total VK usage. Ambiguous assignments accounted for 63.83% of VK genes. JK usage was mainly represented by IGKJ2*01 (35.79%) and IGKJ2*02 (6.15%), with lower contributions from IGKJ1*01 (3.61%) and IGKJ4*01 (0.59%); remaining assignments were not assigned (16.87%) or ambiguous (36.98%).

Importantly, the relative usage of all V, D, and J gene segments remained stable across all time points and did not differ between the total repertoire and the selected clusters, indicating that kinetic cluster selection did not bias global V(D)J gene usage.
