# Supplementary material for: Profiling of porcine B-cell receptor heavy-chain repertoires indicates the development of a wide public pseudorabies virus-specific immune response after vaccination and challenge
Source: Discov Immunol. 2026 May 5;5(1):kyag009. doi: 10.1093/discim/kyag009 (PMC13225268; doi:10.1093/discim/kyag009)
Supplement: kyag009_Supplementary_Data [file kyag009_supplementary_data.zip › FigS5.pdf]

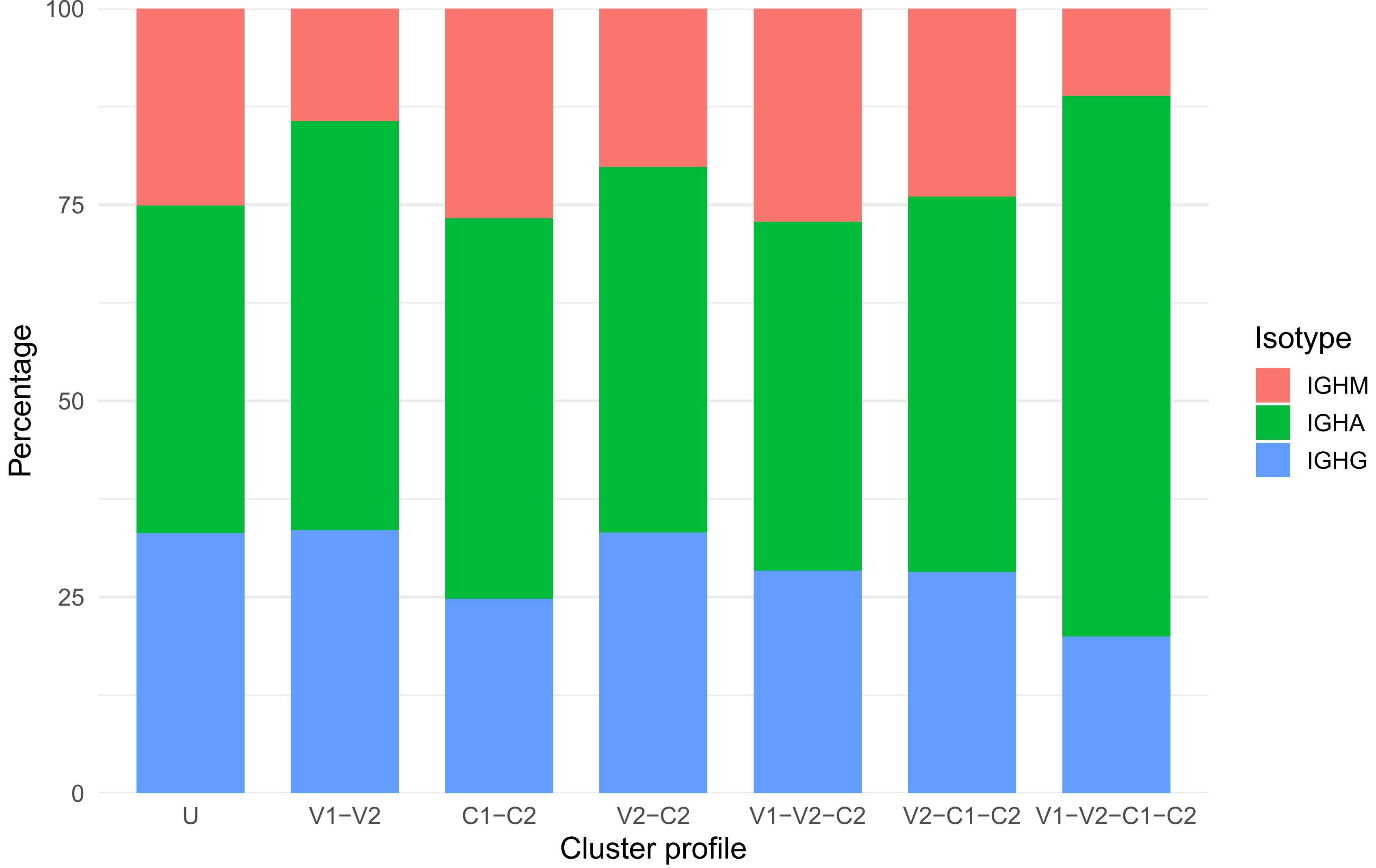

Figure S5. Differences in isotype proportions between PRV-unrelated (U) clusters and selected cluster profiles. The relative proportions of sequenced immunoglobulin isotypes (IGHM, IGHG, and IGHA) were compared across PRV-unrelated (U) clusters and the different selected cluster profiles. Stacked bar plots represent the percentage contribution of each isotype within each cluster profile, highlighting shifts in isotype usage associated with vaccination- and/or challenge-related clusters.
