# Supplementary figures and images for: Profiling of porcine B-cell receptor heavy-chain repertoires indicates the development of a wide public pseudorabies virus-specific immune response after vaccination and challenge
Source: Discov Immunol. 2026 May 5;5(1):kyag009. doi: 10.1093/discim/kyag009 (PMC13225268; doi:10.1093/discim/kyag009)

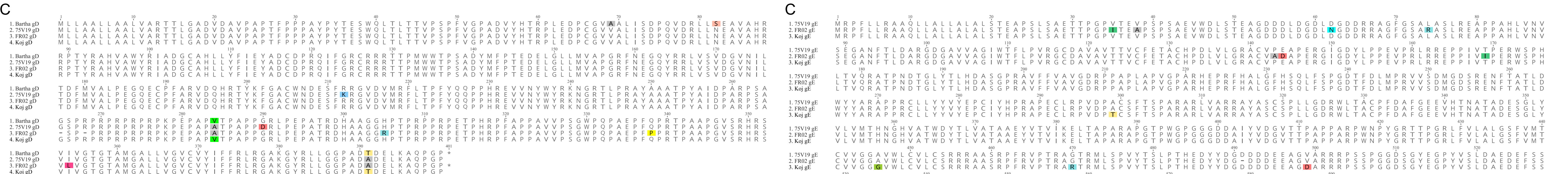

**Figure S1. Amino acid sequence alignment of PRV Kojnok, 175V19, and FR02/075/2016 glycoproteins gB (A), gC (B), gD (C), and gE (D)**

Supplement: kyag009_Supplementary_Data [file kyag009_supplementary_data.zip › FigS1.pdf]
